# High Frequency Deep Brain Stimulation of Superior Cerebellar Peduncles in a Patient with Cerebral Palsy

**DOI:** 10.5334/tohm.551

**Published:** 2020-10-07

**Authors:** Suzhen Lin, Chencheng Zhang, Hongxia Li, Yuhan Wang, Yunhao Wu, Tao Wang, Yixin Pan, Bomin Sun, Yiwen Wu, Dianyou Li

**Affiliations:** 1 Department of Neurology and Institute of Neurology, Ruijin Hospital, affiliated with Shanghai Jiaotong University School of Medicine, Shanghai, CN; 2 Department of Neurosurgery, Center for Functional Neurosurgery, Ruijin Hospital, affiliated with Shanghai Jiaotong University School of Medicine, Shanghai, CN

**Keywords:** Superior cerebellar peduncles, deep brain stimulation, dystonia, cerebral palsy, cerebellum

## Abstract

**Background:**

Globus pallidus internus (GPi) deep brain stimulation (DBS) is widely used in patients with isolated dystonia; however, its use remains controversial in patients with acquired dystonia and cerebral palsy.

**Case presentation:**

We report the first case of a cerebral palsy patient, who failed to recover 2 years after GPi DBS; DBS was administered on both superior cerebellar peduncles (SCPs) and dentate nuclei (DNs). The monopolar stimulation results suggested that DBS was better administered via the SCPs than via the DNs. At six months follow-up, the patient exhibited a significant improvement of dystonia and spasticity, as well as in her quality of life.

**Discussion:**

SCP DBS may be a potential treatment for cerebral palsy patients with dystonia and spasticity who do not respond well to GPi DBS.

## Introduction

Cerebral palsy (CP) is a heterogeneous group of syndromes that vary with regard to the extent of brain damage, etiology, age, and the progression of movement disorders. The most prominent symptoms are dystonia and spasticity. CP constitutes the largest category of acquired dystonia, and the treatment of acquired dystonia in CP is difficult. Pharmacological treatment and surgical therapies such as selective posterior rhizotomy (SPR), intrathecal baclofen infusion, lesional surgery, and deep brain stimulation (DBS) in the internal segment of the globus pallidus (GPi) have consistently shown disappointing results [1,2,3,4]. Due to the lack of a suitable treatment and a good prognosis for CP, other potential treatments need to be explored and developed.

The presence of different etiologies underlies the variable clinical spectrum of CP, but disruption of motor control is almost always reported [5]. Abnormal communication of the cerebellum with the motor cortex, basal ganglia, and/or brainstem can cause involuntary movements such as dystonia, tremor, and spasticity, [6,7] and cerebellar stimulation can affect the pathological abnormalities of this network [8]. Here, we report the case of a CP patient with severe spasticity and dystonia who did not respond well to SPR and bilateral GPi DBS therapy, but has experienced significant improvements after superior cerebellar peduncle (SCP) DBS.

## Case Presentation

A 21-year-old woman with severe fixed dystonia was referred to our hospital for surgical treatment in July 2017. The patient’s medical history showed that she had birth hypoxia. During her first years of life, she presented unwanted movements and dystonic posturing. At the age of 2 years, she had developed generalized dystonia with severe spasticity and was diagnosed with CP. Her symptoms were refractory to medical therapy and SPR.

**Figure 1:**
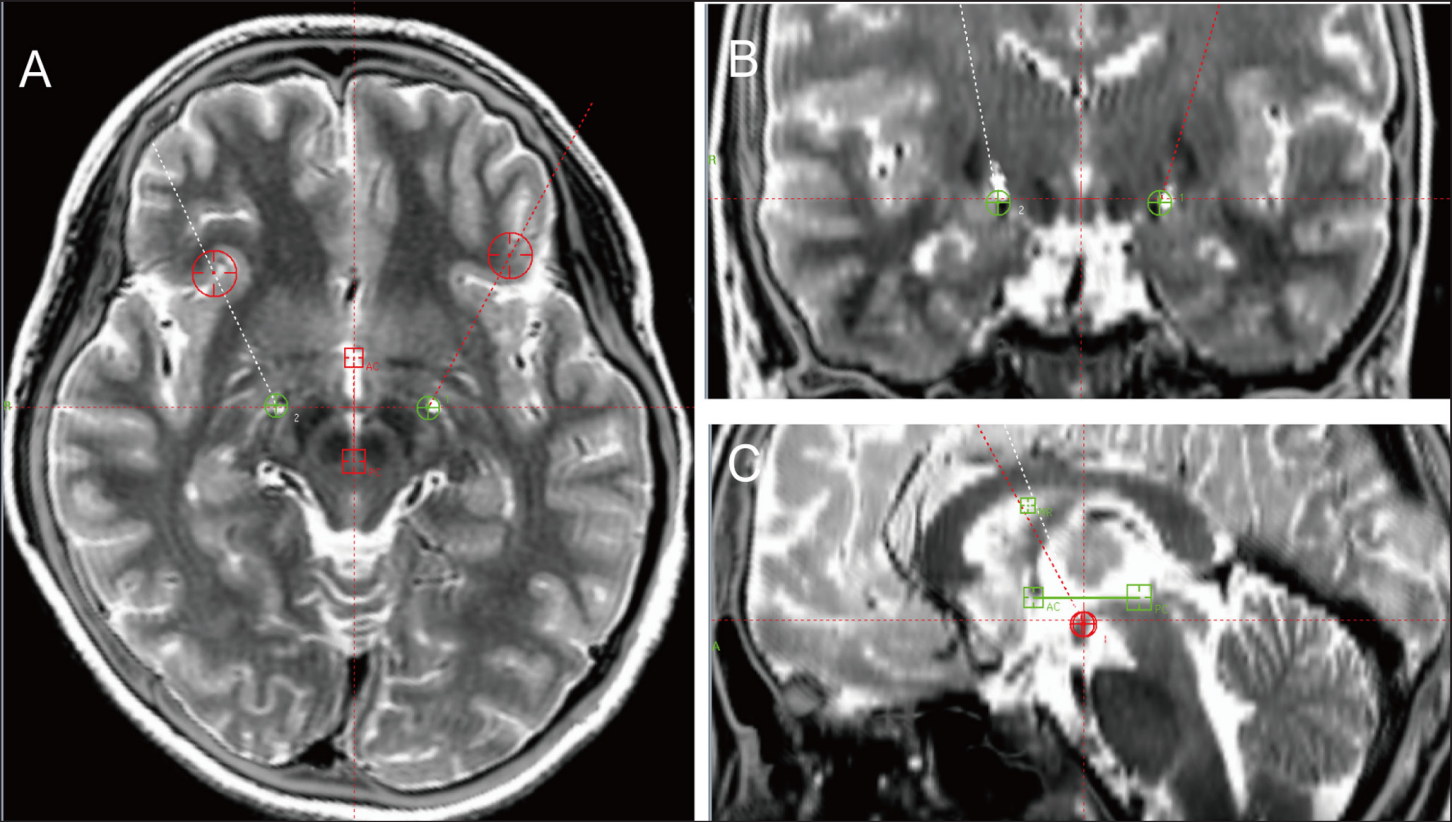
Postoperative CT images fused with preoperative MR images demonstrating the positions of the implanted electrodes in GPi **(1A, 1B, 1C).** The two red orthogonal vertical lines refer to the Cartesian coordinate system in each view, while the two slashes, with or without red circles, present the trajectories of the implanted leads. In the center of each view, two green or red circles show the planned targets. AC, anterior commissure; PC, posterior commissure; MR, midline reference.

She has experienced generalized dystonia with severe spasticity and her speech was also affected. These conditions led her to move using a wheelchair. On admission, she presented with uncontrollable facial expressions and movements, and showed increased muscle tone in the face and the extremities (Figure 4a, Video 1). The patient received GPi DBS in our department in July 2017. GPi DBS were located correctly (Figure 1a–c); however, little stimulation effect was observed after surgery. Her clinical symptoms gradually deteriorated regardless of repeatedly programming (Table 1). At a 2-year follow-up visit, she was unable to sit for prolonged periods of time due to severe spasticity in her trunk and extremities.

**Video 1:** **Pre- and post-video representing the symptoms of the patient**. **Part I:** Symptoms before GPi DBS surgery (video provided by the patient, which was recorded 2 months before GPi surgery). The patient presented with uncontrollable facial expressions and showed increased muscle tone in the face and neck. In addition, the patient can not relex her right leg. **Part II:** Sitting and standing symptoms before cerebellar DBS surgery (2 years after GPi DBS). **Part III:** Sitting and standing symptoms 6months after cerebellar DBS surgery.

**Table 1:** The parameters for GPi DBS of this patient^a^.

DBS site	Follow up time (months)	Stimulation parameters [Amplitude(V)/Frequency (Hz)/pulse width (ms)]

GPi	1	Left:2.3/140/60 Case(+) 1^b^ (–) Right:2.0/140/60 Case(+) 6 (–)
GPi	2	Left:2.95/160/60 Case(+) 1 (–) Right:2.75/160/60 Case(+) 6 (–)
GPi	4	Left:2.45/75/70 Case(+) 1 (–) Right:2.75/75/70 Case(+) 6 (–)
GPi	8	Left:3.65/130/70 Case(+) 1(–)2(–) Right:3.75/130/70 Case(+) 6 (–) 7(–)
GPi	9	Left:2.85/70/70 Case(+) 1 (–) Right:3.0/70/70 Case(+) 5 (–)
GPi	15	Left:3.0/70/70 Case(+) 1 (–) Right:3.25/70/70 Case(+) 5 (–)
GPi	23	Left:3.15/70/70 Case(+) 1 (–) Right:3.45/70/70 Case(+) 5 (–)

^a^ GPi DBS, Globus pallidus internus deep brain stimulation. ^b^ For the electrodes from PINS, the contact numbers 1,2,3,4 are on the left and 5,6,7,8 on the right.

In July 2019, the patient consulted our hospital again for further treatment. Upon admission, the patient presented with involuntary movements in multiple body parts including eyes, mouth, neck, trunk, and all four extremities (Figure 4b and c, Video 1). During examination, muscle spasms were found in all four extremities, especially in the left arm, right leg, flexion of the left elbow, and right knee. Due to truncal dystonia, the patient was unable to stand and needed a back support while sitting. In addition, the patient’s speech was laborious, non-fluent, and incomprehensible (Table 3 and Figure 4f). The patient also experienced pain in the extremities (visual analog scale score = 6; Burke-Fahn-Marsden dystonia rating scale movement sub score [BFMDRS] = 93.5; and the disability sub score = 21). The modified Ashworth scale (MAS) for rating the spasticity was three and four for the right arm and other limbs, respectively. The score for quality of life using the 36-item short form health survey (SF-36) was 123, and moderate depression was revealed by a Beck Depression Inventory score of 14.

**Figure 2:**
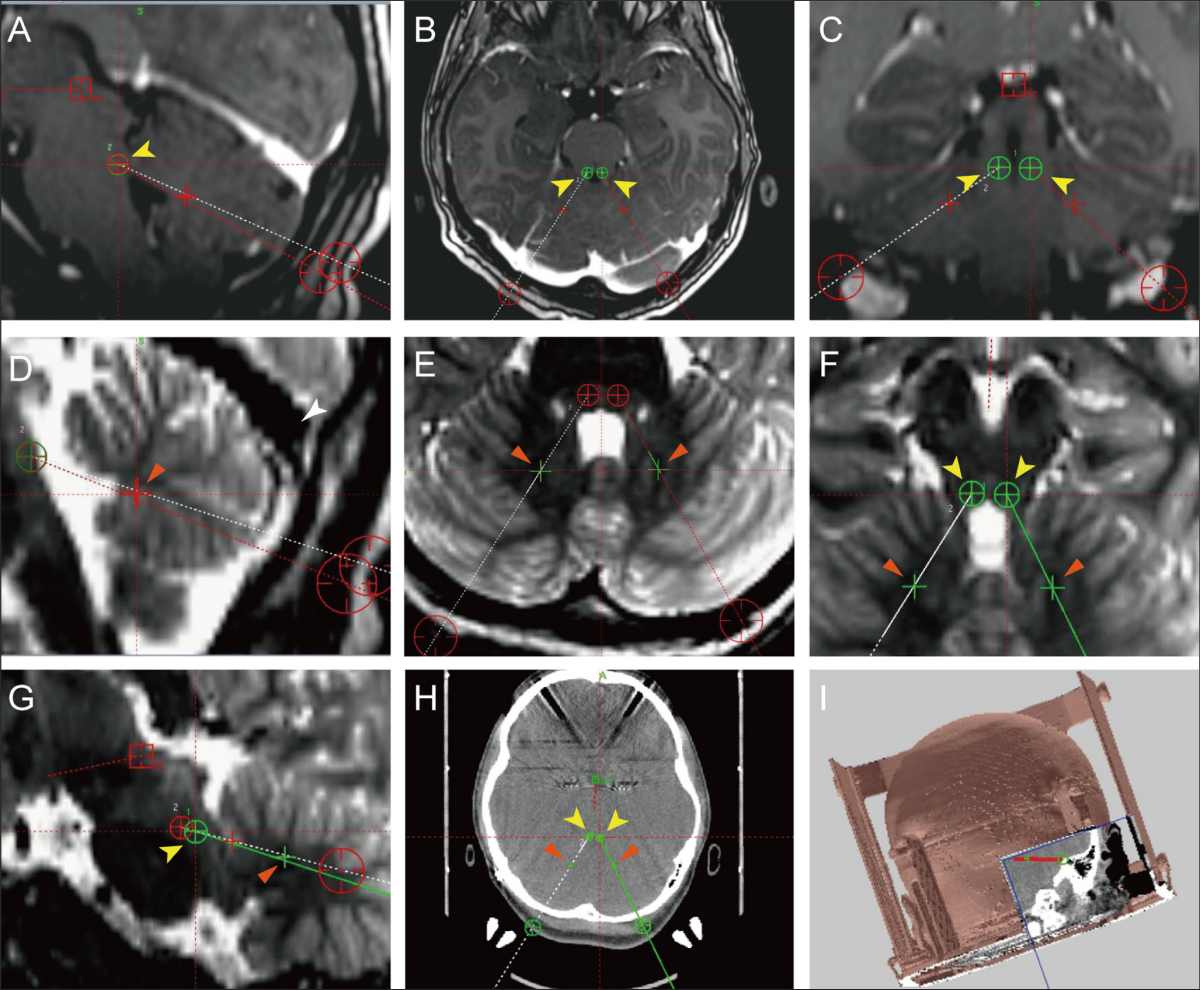
**Determining the coordinates of the target superior cerebellar peduncles (SCPs) and dentate nuclei (DNs).** The yellow arrows are directed to the SCPs, and orange arrows are directed to the DNs. The stereotactic coordinates of the SCP were identified on the sagittal section as the end point of the SCP to the brainstem (z) **(2A)** in line with the floor of the fourth ventricle on the horizonal section (y) **(2B)** and 3 mm lateral from the midline of the coronal section (x) **(2C).** Coordinates of the DN region were directly under the fastigium of the fourth ventricle on the mid-sagittal section (z, y) **(2D)** and 13 mm lateral from the midline on the T2 horizonal image **(2E).** The trajectory was planned using SCPs as the primary target. The coronal and sagittal angles were adjusted to attain a trajectory that traversed the origin of the SCP and target the DN. The trajectory was identified in the horizonal **(2F)** and sagittal sections **(2G)**, and the entry point was 1 cm away from the sigmoid sinuses in the MRI image and at least 1 cm away from the posterior fixation posts and posterior frame bar in the CT image **(2H).** The 3D trajectory is shown in **(2I).**

**Figure 3:**
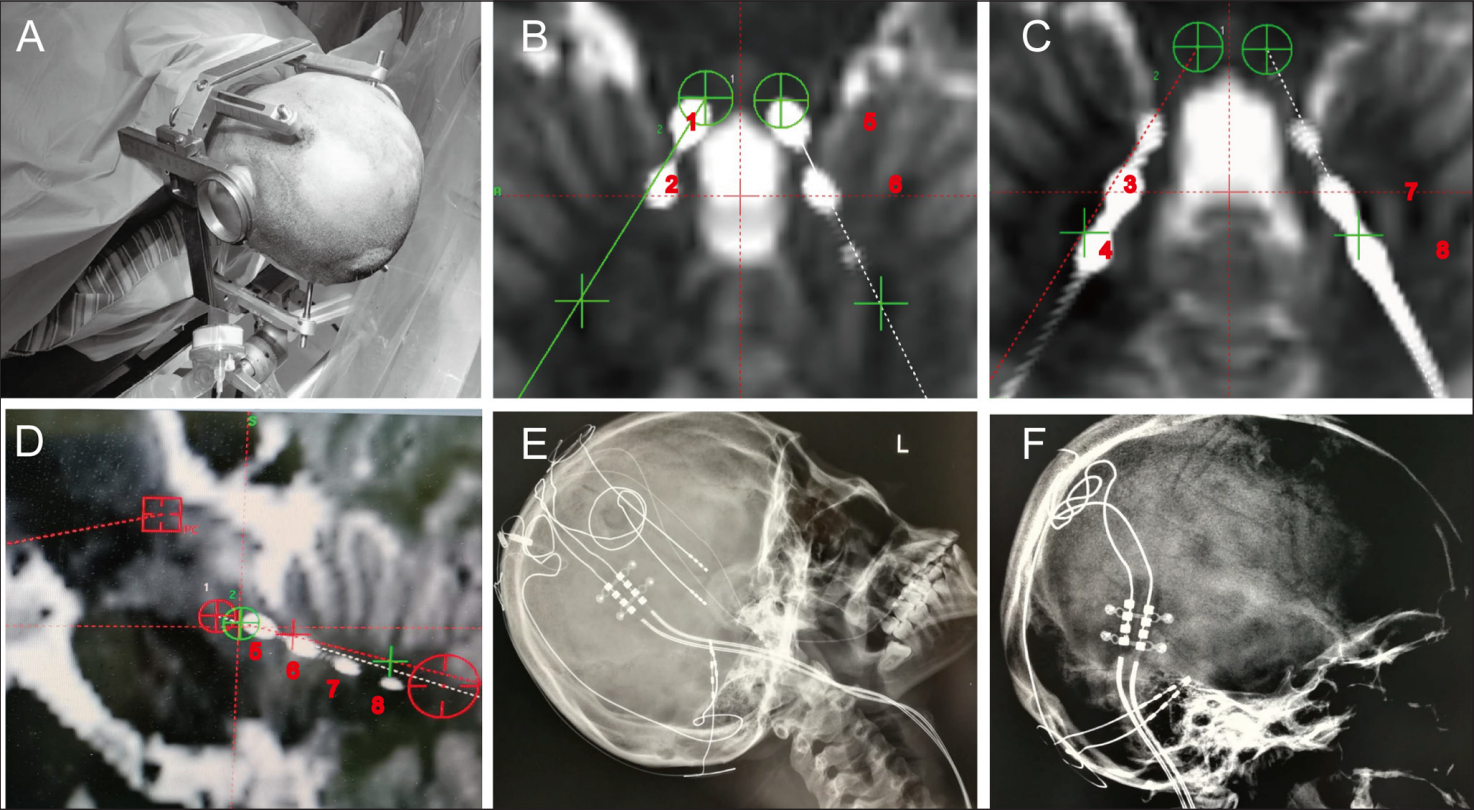
**Images showing the surgical procedure as well as the postoperative head images.** During the first stage of trial stimulation, a stereotactic frame (Leksell) was installed under local anesthesia, and head computed tomography (CT) scan (Helex Sequence) was performed. Given the location of the target and the suboccipital approach, the head was fixed to the bed with the straight front piece of the Mayfield head rest during prone position for the anterior bar of the frame, and the long posterior fixation posts, which make the stereotactic frame, were placed lower than usual **(3A).** For the contacts of the leads to traverse both the SCP and DN, the customized DBS lead for research (L303, Beijing Pins Medical Co., Ltd., Beijing, China) was used. The lead featured 4.0 mm spacing between each of the four 3.0 mm electrodes at the distal end; the electrode spread is 24.0 mm with the ventral contact within SCP and the dorsal contact within DN. For the operation, two L303 DBS leads were implanted into the patient in the prone position under general anesthesia according to the planned coordinates and trajectory with the lead connected to the external extension wire for trial stimulation. The bilateral GPi leads were retained during this step of surgery **(3E).** After 1 week of trial stimulation, we removed the bilateral GPi DBS leads **(3F)** and connected the SCP DBS leads to the previous implanted pulse generator (G102R, PINS, Beijing Pins Medical Co., Ltd., Beijing, China). A 1.5 T T1- and T2-weighted MRI is allowed for patients with implanted DBS leads according to the manufacturer’s indications. Ventral contacts 1 (or 5 on the left side) and 2 (or 6) were close to the superior cerebellar peduncle (SCP) **(3B)**, and dorsal contacts 3 (or 7) and 4 (or 8) were close to the dentate nuclei (DN) **(3C).** All four contacts of the left side are shown in **(3D).** The X-ray image **(3E)** showed both the new implanted electrodes in the cerebellum and the old electrode in the globus pallidus internus (GPi), while the **(3F)** showed only the new implanted electrodes in the cerebellum existing after explanted the old electrodes in GPi.

Due to limited treatment options and the potential role of the cerebellum in dystonia and spasticity, [9] we proceeded with cerebellar DBS. The ethics committee of the Shanghai Jiaotong University School of Medicine approved this procedure. Written informed consent was obtained from the patient and her parents. The surgical procedure is described in detail in Figures 2, 3 legends and the MRI images for determining the coordinates of the target SCPs and DNs are shown in Figure 2.

## Results

### Post-operative course

Two days after surgery, postoperative CT was computationally fused with preoperative MRI to confirm the expected lead placement (Figure 3). As shown in Figure 3, ventral of contact 1 (or 5 on the left side) and 2 (or 6) were in close proximity to the SCP, and dorsal of contact 3 (or 7) and 4 (or 8) were in close proximity to the DN.

The monopolar test stimulation of every contact was done at a frequency of 130 Hz, pulse width of 60 µs, and gradual increments of voltage from 1 V to 10 V beginning at 1 V in each contact. The increase in voltage was terminated when patients experienced intolerable side effects. In addition, voltage was reduced to 0 before the subsequent stimulation to test the sudden response of each stimulation intensity (Table 2 shows the detailed reactions of the stimulation). Contact 1 (or 5) induced an increase in muscle tone on the ipsilateral limbs for several minutes followed by immediate relaxation. Concurrently, the patient experienced paresthesia on the contralateral side of her face and the ipsilateral limb that she managed to endure for up to 3 V before the pain was unbearable. Similar reactions were observed for contact 2 (or 6), except that the patient’s head and gaze were turned toward the ipsilateral side at 4 V stimulation. For contact 3 (or 7) and 4 (or 8), higher voltage was needed to detect a reaction, and improvements of symptoms were always accompanied by significant side effects including dizziness, nystagmus, and ipsilateral leaning. Therefore, stimulation of contact 1 (or 5) and 2 (or 6) had the best therapeutic response and the least severe side effects. The optimal settings were 1+, 2–, and 2.45 V for the right side; and 5+, 6–, and 1.85 V for the left side, with a pulse width of 60 µs and a frequency of 130 Hz.

**Table 2:** Detailed information and reactions of the patient during the testing of each contact at 2-day follow up.^a^

Position	Contact	Frequency (Hz)	Pulse width (µs)	Voltage (V)	Affected body parts	Side effects

Right	1	130	60	2	con.face	
				3	ip.leg; con.face	pain in con.face
Right	2	130	60	2	con.face	
				3	ip.leg; con.face	
				4	ip.leg; con.face	gaze deviation; lower speech tone
				5	ip.leg; con.face	ip.leaning
Right	3	130	60	3	con.face	pain in con.face
				4	ip.leg; con.face	Gaze deviation; dizziness; ip.leaning; nystagmus
Right	4	130	60	8	con.face	Gaze deviation;

Left	5	130	60	1	ip.leg; con.face; con.arm; con.finger	
				2	con.face	pain in con.face
				3	con.face	pain in con.face
Left	6	130	60	2	con.face; ip.leg	
				3	con.face; ip.leg	head ip.leaning
				4	con.face; ip.leg	head ip.leaning; pain in con.face
Left	7	130	60	2	ip.leg	
				3	ip.leg	head ip.leaning
				4	ip.leg	head leaning; dizziness
Left	8	130	60	7	ip.leg	
				8	ip.leg	ip.leaning

^a^ Reaction in face was tingle; reaction in limbs was increases in muscle tonus accompanied by immediate relaxation. The first voltage number listed for each contact is the minimum voltage that causes patient’s reaction. Pain in face means the patient cannot tolerate the tingle and yell. ip, ipsilateral; con, contralateral; ip.leaning means the whole body including head and trunk lean to the ipsilateral side; head ip.leaning means only head leans to the ipsilateral side.

**Table 3:** Pre- and post-operative clinical scores following SCP DBS in a patient with CP.

	Scales	Subscale	Pre-op	3 months post-surgery	6 months post-surgery

	MAS	Upper extremities (right)	3	2	1
		Upper extremities (left)	4	2	2
		Lower extremities (right)	4	4	4
		Lower extremities (left)	4	3	3
	BFMDRS	Movement scores	92	66.5	59.5
		Disability scores	21	15	14
	VAS		6	1	0
	SF-36	Physical function	0	15	20
		Role, physical	0	75	100
		Role, emotional	0	100	100
		Body pain	41	74	84
		Mental health	32	52	52
		General health	25	55	65
		Social function	0	50	50
		Vitality	25	65	70
	BAI		9	2	2
	BDI		14	6	4
	MoCA		18	24	23
Voice	Quality	Basal frequency (Hz)	192.11	212.77	219.38
		Jitter (%)	1.12	0.58	0.54
		Shimmer (dB)	4.948	3.39	2.988
		Noise-harmonic ratio	0.1822	0.114	0.0878
		Breath Sound Index	0.6	2.08	2.15
		Irregularity	1.72	1.4	0.81
		Maximum phonation time (s)	3.56	7.36	9.12
		DSI index	–0.2	0.8	2.9
	Articulation	Jaw distance	550.73	535.92	508.96
		Tongue distance	1283.79	649.92	1783.17
		Vowel space area (VSA)	281914	278951	372958
	Fluency	DDK rate (syl/s)	2.82	3.17	4.38
	Self-value	Function	2	0	0
		Physiology	11	4	2
		Emotion	12	4	1

SCP DBS, superior cerebellar peduncle deep brain stimulation; CP, cerebral palsy; MAS, Modified Ashworth Scale; BFMDRS, Burke-Fahn-Marsden Dystonia Rating Scale; VAS, visual analog scale; SF-36, 36-item Short Form Health Survey; BAI, Beck Anxiety Inventory; BDI, Beck Depression Inventory; MoCA, Montreal Cognitive Assessment.

### Outcomes

At the 3-month follow up visit, the BFMDRS showed an improvement of 28.9% (pre-operation = 93.5, post-operation = 66.5), with considerable improvements in activities using the upper extremities, such as brushing teeth and combing by herself. The patient’s voice quality, speech fluency, and self-esteem improved. Notably, the stimulation parameters were reduced to 1+, 2–, and 2.35 V for the right side and 5+, 6–, and 1.75 V for the left side, with a reduced pulse width of 50 µs and a frequency of 130 Hz. The patient was able to sleep much better during nighttime than before surgery. Hence, we set a daily cycling mode with stimulation from 6:30 am till 23:30 pm. Four months after SCP stimulation, the patient presented in a television show.

**Figure 4:**
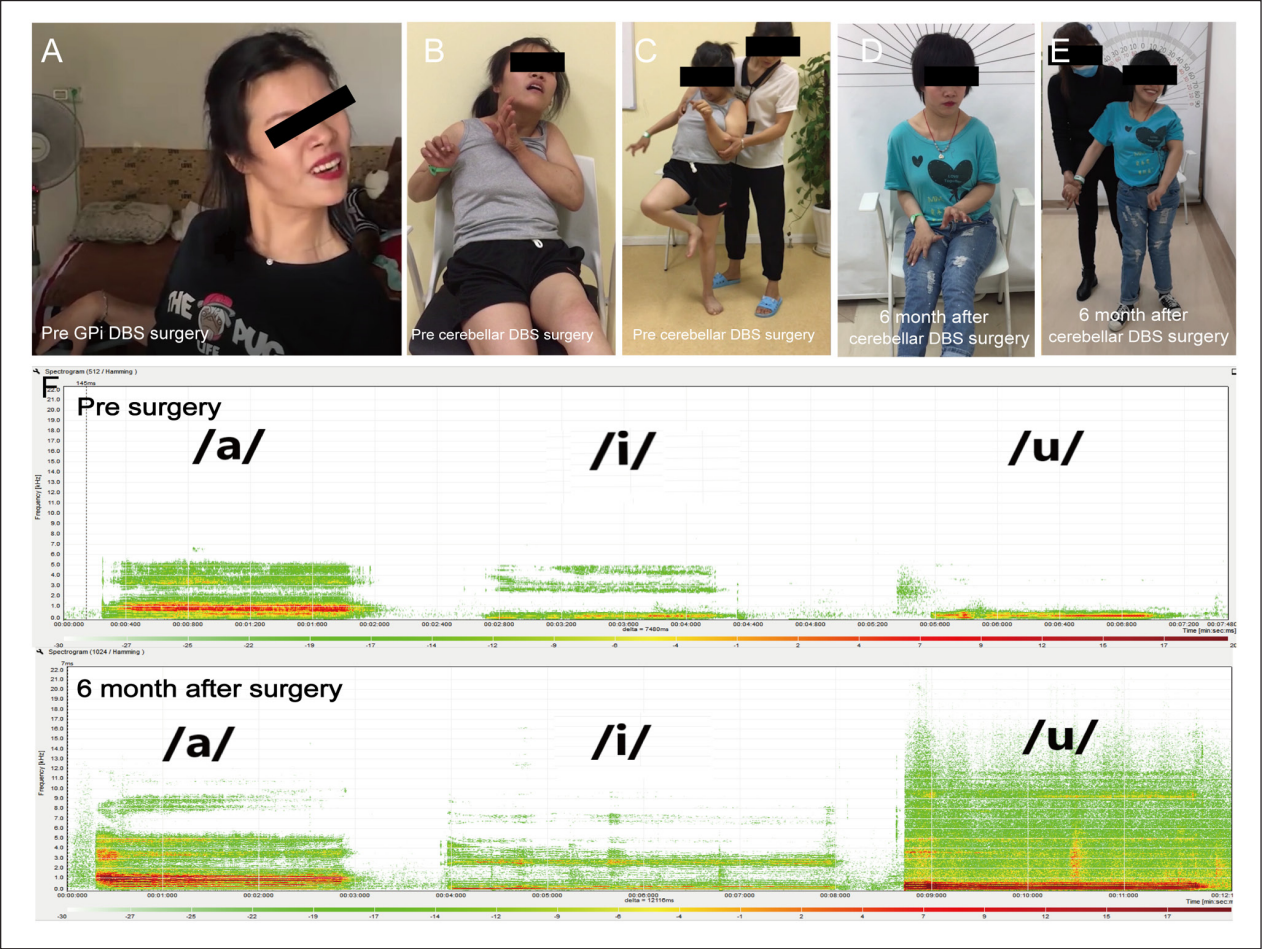
**Pre- and post-images representing the symptoms of the patient. (4A)** Photograph recorded before GPi DBS surgery. Pre-**(4B)** and **(4C)** and post-operation-**(4D)** and **(4E)** (cerebellar DBS) photographs of the patient’s general appearance on standing and sitting. **(4F)** Vocal spectrum before and after the operation.

At the 6-month follow up, the BFMDRS showed an improvement of 36.4% (pre-operation = 93.5, post-operation = 59.5) with considerable improvements in the upper extremities, axial dystonia, and speech, and a slight improvement in the lower extremity dystonia (Figure 4d–e). The patient’s disability scale score reduced to 14, with improvements primarily in her hand and speech. She was able to write and apply makeup for herself, tasks which were impossible before the surgery. Moreover, the patient was able to send voice messages in WeChat. The MAS scores of the upper limbs (right, left) and lower limbs (right, left) post-operation were 1, 2, 4, and 3, respectively, while at pre-operation they were 3, 4, 4, and 4, respectively (Table 3). Notably, the patient was able to sit without a back support and to stand on both legs (Video 1).

The SF-36 scale results showed a significant improvement in her quality of life at both the 3- and 6-month follow up. The vocal analysis indicated a much clearer and more fluent speech after surgery. Five months after surgery, the patient hosted a live show on a Chinese network platform. The stimulation parameters were the same as the 3-month follow up with no significant perioperative or hardware-related complications.

## Discussion

The present case report demonstrated the ability of SCP DBS to reduce pain, dystonia, and spasticity, and to improve the quality of life in a patient with CP who did not respond well to GPi DBS. Our results confirmed the beneficial effect of DBS administered via the SCP, which links the DN to cortical and subcortical structures.

Recently, evidence of the connections between the cerebellum and basal ganglia has led to a resurgence of interest in the cerebellum involvement in modulating motor functions. The dentato-rubro-thalamic and cerebello-thalamo-cortical tracts are two well recognized pathways that could account for the movement regulatory mechanism of the cerebellum [10]. The cerebellum is believed to directly modulate the basal ganglia, and abnormal cerebellar output has been reported to cause movement disorders [6,11]. Therefore, many research groups have started to reconsider the efficacy of cerebellar interventions for several disorders including CP.

Before the widespread use of DBS, chronic cerebellum stimulation had long been used to treat patients with CP, the majority of whom showed improvements in dystonia and spasticity. Cooper et al. first treated patients with CP using cerebellum stimulation of the anterior lobe (which contains the SCP) in 1973 [12]. Rose Davis et al. [9] and Galanda et al. [13,14,15] have reported that cerebellar anterior lobe stimulation reduced the severity of spasticity in patients with CP.

DBS is conducted by a cylinder lead that can be precisely inserted into deep brain targets such as the DN and SCP using a stereotactic technique. Recently, case reports have demonstrated the efficacy of DN or SCP DBS in patients with dystonia [16,17,18]. However, there is limited clinical evidence for the efficacy of cerebellar DBS, and consensus on the selection of appropriate stimulation targets for patients with CP has yet to be reached. In this case, we targeted both SCP and DN with a single DBS, and both targets alleviated the patient’s symptoms. Unfortunately, DN stimulation needed stronger stimulation intensity and was accompanied by several side effects including dizziness, nystagmus, and ipsilateral leaning compared to the SCP target. Shiro Horisawa et al. also reported a patient with generalized dystonia who responded well to SCP DBS than DN DBS [16]. The study reported by Brown et al. showed the validity of DN DBS in treating hemidystonia; however, it is worth noting that the active contacts were in close proximity to or within SCP [17].

DN and SCP are both important nodes in the cerebellum pathways that are connected to the brain. The DN receives ascending projections from the spinocerebellar tract as well as the premotor and supplementary motor cortices and integrates them. Its efferent projections travel through the SCP to the red nucleus, the thalamus, the basal ganglia, and other structures and subsequently transfer modulating signals to those regions [19]. Thus, the proximity of SCP to the downstream regions of DN may be of advantage for SCP stimulation to exert a better effect than DN stimulation but further examination is needed to confirm this. In addition, DN is the largest deep cerebellar nuclei that is approximately 13 mm to 23 mm in length, 7 mm to 20 mm in height, and 9 mm to 20 mm in width, [20] which is significantly larger than a DBS electrode [21]. The deviation of electrode placement in DN stimulation due to the large size of the DN may account for the variable effects observed in those reported cases and in our case.

The stimulation parameters (1.85~2.45 V, 50 µs, 130 Hz) used in this study were lower than most previously reported stimulation parameters of cerebellar DBS studies (Table 4) [13,14,15,16,17,18]. The selection of the electrode lead and correct lead placement may be the key step for the low parameter in our study. In a previous report, [16] a 3387 lead (Medtronic, 1.5 mm contacts) was used and a very high stimulation (8.0 V, 150 µs, 200 Hz) was needed to achieve any clinical effect. Here, we selected an electrode with four longer lead contacts (3 mm), which may result in a much larger stimulation area and allow stimulation at increasing intensity and density [22]. In addition, our contacts on the SCP were near the midbrain, and electrodes were positioned across both the SCP and DN to allow optimal target programming. The active contacts within SCP were about 10 mm in length, thus 3387 lead alone may also be enough for SCP stimulation. Future studies should test the potential advantages of SCP in treating dystonia and address the underlying mechanisms.

**Table 4:** The list of all the reported simulation parameters and lead model for cerebellar stimulation^a^.

Authors	Galanda et al	Galanda et al	Galanda et al	Sokal et al	Taira et al	Brown et al

Year	1997	2003	2007	2015	2019	2019
Number of cases	31	3	4	10	1	1
Disease	Cerebral palsy	Cerebral palsy	Cerebral palsy	Cerebral palsy	Generalized dystonia	Acquired hemidystonia
Target	SCP	Anterior lobe of the cerebellum	Anterior lobe of the cerebellum	Deep region of anterior lobe	SCP and DN	DN
Lead model	TESLA LSP 330; Minneapolis, Medtronic	Model 3387, Medtronic	Model 3387, Medtronic	/	Model 3387, Medtronic	Model 3387, Medtronic
Contacts	/	/	/	/	0 to 1–/3+	1–2–3+
Frequency (Hz)	200 or 130	185	185-200	130	200	130
Pulse width (ms)	/	210	210	150-180	150	60
Voltage (V)	1~6	0.5~4	0.5~2.5	1.4~2.4	8	1.2~2.8
Reactions by threshold stimulation	Pronounced pathological posture; dyskinesias; Fear;	Unpleasant fear	Intense feeling of pleasure; increase in muscular tone; unpleasant fear; immediate relaxation and reduction of spasticity.	/	/	Leaning and head bobbing; Vocal impairment; appendicular ataxia; titubation.

^a^ SCP, Superior cerebellar peduncle; DN, Dentate nucleus.

Although our study has demonstrated the potential role of SCP DBS in improving the refractory symptoms of dystonia and spasticity in CP compared to conventional treatment, these results should be interpreted with caution. First, our patient had a history of hypoxia at birth, which may result in altered brain anatomy, though the cerebellum appeared intact on MRI. Second, the patient experienced SPR and GPi DBS, which might have altered he anatomy of her nervous system. In addition, this study only involved one patient and the etiology, age, and movement disorder progression can vary greatly between patients. Therefore, further studies are needed to elucidate the effects of SCP DBS for patients with CP.
